# Bipolar disorder in youth is associated with increased levels of vitamin D-binding protein

**DOI:** 10.1038/s41398-018-0109-7

**Published:** 2018-03-13

**Authors:** Brawnie Petrov, Ayat Aldoori, Cindy James, Kefeng Yang, Guillermo Perez Algorta, Aejin Lee, Liwen Zhang, Tao Lin, Reem Al Awadhi, Jonathan R. Parquette, Arpad Samogyi, L. Eugene Arnold, Mary A. Fristad, Barbara Gracious, Ouliana Ziouzenkova

**Affiliations:** 1 Human Nutrition, The Ohio State University, Columbus, OH 43210 USA; 2 Mass Spectrometry and Proteomics Facility, The Ohio State University, Columbus, OH 43210 USA; 3 Department of Nutrition, School of Medical, Shanghai Jiao Tong University, Shanghai, 200025 China; 4 Division of Health Research, Spectrum Centre, Lancaster University, Lancaster, LA1 4YW UK; 5 Department of Chemistry and Biochemistry, The Ohio State University, Columbus, OH 43210 USA; 6 Department of Medicine and Surgery, Royal College of Surgeons in Ireland, Dublin 2, D02 Y651 Ireland; 7 Department of Psychiatry and Behavioral Health, The Ohio State University, Columbus, OH 43210 USA; 8 Department of Child and Adolescent Psychiatry and Behavioral Health, Nationwide Children’s Hospital, Columbus, OH 43205 USA

## Abstract

Genetic, dietary, and inflammatory factors contribute to the etiology of major mood disorders (MMD), thus impeding the identification of specific biomarkers to assist in diagnosis and treatment. We tested association of vitamin D and inflammatory markers in 36 adolescents with bipolar disorder (BD) and major depressive disorder (MDD) forms of MMD and without MMD (non-mood control). We also assessed the overall level of inflammation using a cell-based reporter assay for nuclear factor kappa-B (NFκB) activation and measuring antibodies to oxidized LDL. We found that these factors were similar between non-mood and MMD youth. To identify potential biomarkers, we developed a screening immunoprecipitation-sequencing approach based on inflammatory brain glia maturation factor beta (GMFβ). We discovered that a homolog of GMFβ in human plasma is vitamin D-binding protein (DBP) and validated this finding using immunoprecipitation with anti-DBP antibodies and mass spectrometry/sequencing analysis. We quantified DBP levels in participants by western blot. DBP levels in BD participants were significantly higher (136%) than in participants without MMD (100%). The increase in DBP levels in MDD participants (121.1%) was not statistically different from these groups. The DBP responds early to cellular damage by binding of structural proteins and activating inflammatory cells. A product of enzymatic cleavage of DBP has been described as macrophage-activating factor. Circulating DBP is comprised of heterogenous high and low molecular fractions that are only partially recognized by mono- and polyclonal ELISA and are not suitable for the quantitative comparison of DBP in non-mood and MDD participants. Our data suggest DBP as a marker candidate of BD warranting its validation in a larger cohort of adolescent and adult MMD patients.

## Introduction

Major mood disorders (MMD), specifically bipolar disorder (BD) and major depressive disorder (MDD), are some of the most prevalent albeit under-diagnosed health problems in children and adolescents^1,2^. Worldwide among adolescents, MDD and BD are the first and fourth most disabling conditions, respectively^3^. High mortality in adults with MDD and BD is attributed to suicide and cardiovascular-related disorders^4^. Currently, there is an estimated 10-year delay between onset of BD and accurate diagnosis^5^ because robust biomarkers for MMD have not been identified.

Biomarker discovery is impeded due to the insufficient understanding of the underlying etiology and pathophysiology of MMD^6^. Pathophysiology of BD has been attributed to deficits in serotonin^7^. Microglia macrophages produce and sense serotonin in response to proinflammatory cytokines in the brain and peripheral nervous system^7^. MDD has been associated with a reduction in glial cell counts and density compared to healthy controls^8^. In contrast, activated microglia release proinflammatory cytokines in BD, thereby, exerting negative effects on the neuroprotective system and mediating further pathophysiological disturbances^9^. Glia maturation factor beta (GMFβ), which is expressed in cerebral astrocytes, activates the microglia^10^. Activated microglia release proinflammatory cytokines, which exert negative effects on the neuroprotective system and mediate further pathophysiological disturbances related to BD^9^. These disturbances include alterations in synaptic function as well as in apoptosis, excitotoxicity, and downregulation in neurogenesis and neurotrophin production^9,11^. It still unclear what processes trigger inflammation in the brain, but inflammatory signaling in the kynurenine pathway, an alternate route of tryptophan metabolism that decreases serotonin neurotransmission, as well as activation of the enzyme glycogen synthase kinase-3 beta (GSK-3β) in the Wnt pathway, and activation of cyclooxygenase 2 and arachidonic acid appear to be involved^11^.

Given that inflammation influences abnormalities in glia and neuronal plasticity^7^, systemic inflammation has also been proposed as a mechanism or a result of MMD depending on etiologies^9,12–14^. Elevated cytokine levels in peripheral blood and cerebrospinal fluid, and altered inflammatory activity are found in those with MMD compared to non-depressed individuals^12,13^. Specifically, tumor necrosis factor-alpha (TNF-α) is higher in adult patients with MDD^15^. Cytokines, such as interleukin (IL)-4, IL-6, and IL-8, are also reportedly altered in patients with MDD^16,17^. The elevated levels of proinflammatory cytokines in patients with MDD, who are otherwise healthy, suggest a likely link between depressive illness and dysregulation of the inflammatory response^15,18,19^. In addition to elevated proinflammatory cytokines, BD is also associated with hyperactivity of T-helper cell 1, with significantly higher levels in BD patients during manic and depressive episodes as compared to non-BD controls^12,20,21^.

Nuclear factor kappa-B (NFκB) is a key transcription factor in the regulation of increased expression of proinflammatory cytokines and neuroendocrine responses to stress^22,23^. Hyperactivation of NFκB in BD is also thought to be a protective mechanism against the neurotoxic effects of repeated illness episodes^22,24^. Despite the evidence that the inflammatory cascade is regulated by activation of NFκB^23,25,26^, few studies have examined NFκB regulation in MMD in pediatric-onset MDD or BD^22^. Manifestations of inflammation in studies of youth with MMD vary, and to date no robust marker predicting onset of MMD in an adolescent population has been reported.

The relatively new field of psychiatric nutrition has highlighted a relationship between diet and the mental health status of children and adolescents^27–31^. Vitamin D has been shown to influence inflammation-dependent disorders of the central nervous system (CNS) in multiple sclerosis^32,33^, amyotrophic lateral sclerosis^34^, and MMD^35^. Gracious et al.^36^ reported an association between vitamin D deficiency in adolescents and severity of mental illness. A pilot study of youth with BD documented both improvement of manic and depressive symptoms and normalization of brain neurochemistry via neuroimaging following an 8-week open-label trial of 2000 IU of vitamin D3^37^. Vitamin D may participate in the regulation of brain function, as vitamin D receptors (VDR) are present in large amounts in the CNS and in the developing and mature human brain^38,39^. Vitamin D is transported by vitamin D-binding protein (DBP), but little is known about its role in the CNS. DBP is expressed in the rat^40^ and possibly in human brain^41–43^. DBP is found in human cerebrospinal fluid^32,44^.

DBP has three domains, with each serving a specific function^42^. Vitamin D vitamers are bound in the first domain^42^. Only 1–2% of DBP are bound to vitamin D, suggesting that DBP function extends beyond its vitamin D transport properties^41,43^. The second and third domain of DBP binds fatty acids^42,45^, extracellular structural proteins^42,46^, and trombospondin^47^, in the circulation after cell necrosis and tissue injury. The carboxy-terminal domain of DBP also contains an O-linked glycosylation site^48,49^. Enzymatic cleavage of this side transforms DBP into a lower molecular weight DBP-L (53.4kD)^48^. This DBD-L was characterized as a potent macrophage-activating factor (termed DBP-MAF)^49^. The potential relevance of DBP modifications to MMD pathogenesis is unknown.

In this pilot study, we tested potential blood-based markers of inflammation, NFκB activation, and vitamin D in adolescents with MMD, to better understand their contribution to MMD in youth^4,14,22,50^. Using a new strategy, we identified a candidate biomarker for BD that changes in response to inflammation, cell death, and vitamin D status.

## Methods

Detailed description of studies, experimental procedures, and reagent information is provided in Supplementary Materials.

### Patients

Plasma samples used in this study were collected from a subset of participants enrolled in the National Institute of Mental Health (NIMH) Longitudinal Assessment of Manic Symptoms (LAMS) study, which examines mood symptom changes and elevated symptoms of mania (ESM) biannually in 685 youth aged 6–12 years recruited at four sites^51^ The Ohio State University (OSU) Institutional Review Board approved both the LAMS study and this sub-study. Assent from the youth and informed consent from the parent were obtained prior to data collection.

### Study design and clinical assessment

Clinical study procedures were conducted in the Child Mood Lab of Dr. Fristad at the OSU Wexner Medical Center Harding Hospital. Diagnostic data used in this study were obtained by post-doctoral or graduate student trained interviewers who met ongoing standards for inter-rater reliability. Mood questions from the Kiddie Schedule for Affective Disorders and Schizophrenia for School-Age Children-Present and Lifetime Episode (K-SADS-PL-W) were used to determine MMD. Interviewers were unaware of vitamin D level results at the time of the interviews.

Participants (*n* = 36) were grouped into three categories: Non-mood controls (*n* = 13), bipolar (BD) (*n* = 12), and MDD (*n* = 11). Groups were assigned based on consensus conference review led by a licensed child/adolescent psychologist. Body mass index was calculated using standardized height and weight measures. Blood was collected by OSU Clinical Research Center nursing staff.

Concentrations of inflammatory cytokine IL-6, autoantibodies to oxLDL, and vitamin D in serum as well as serum NFκB activity were measured and analyzed in conjunction with metabolic characteristics (body mass index (BMI)). All experiments except clinical vitamin D levels were performed in a ‘‘blind’’ fashion using coded information from participants.

### Vitamin D detection

Vitamin D (25-hydroxycalciferol and 25-hydroxyergocalciferol) analysis on serum was performed by high-pressure liquid chromatography (HPLC) coupled with mass spectrometry (MS) detection at the Esoterix Laboratory Services, Inc. (Austin, Texas).

### NFκB assay

The cumulative NFκB activation potential of plasma was measured using NF-κB/green fluorescence protein biosensor assay as described previously^52^ and in Supplementary Materials.

### Immunoprecipitation and LTQ Orbitrap

Mouse anti-human GMFβ antibody (Proteintech, Rosemont, IL, USA) was conjugated on beads (Millipore, Darmstadt, Germany). Cell lysates were incubated with anti-GMFβ-conjugated beads according to manufacturers’ protocol. Proteins bound to anti-GMFβ-conjugated beads were eluted, digested, and identified by capillary-liquid chromatography-nanospray tandem mass spectrometry (Capillary-LC/MS/MS) of global protein identification was performed on a Thermo Fisher LTQ orbitrap mass spectrometer equipped with a microspray source (Michrom Bioresources Inc, Auburn, CA) operated in positive ion mode. One-hundred twenty-six proteins were identified by immunoprecipitation using anti-GMFβ antibody that were present at higher levels in serum from MMD than non-mood control group. We excluded proteins that were not present in plasma in one group of patients (Ratio 0 or n/a). The size of putative GMFβ-positive homolog (50 ± 5 kD) was determined using individual plasma samples from all patients analyzed by western blot using the same antibodies. Homology comparison was performed for proteins that had a molecular weight of 50 ± 5 kD. The remaining protein structures were analyzed for the homology to GMFβ (Accession number NP_004115.1) using NCBI BLAST program. DBP accession number used for this program was AAA61704.1.

A mouse monoclonal antibody raised against amino acids 175–474 mapping at the C-terminus of DBP of human origin (Santa Cruz Biotechnology, sc-365441) was conjugated on beads and immunoprecipitation was performed as described above. Samples were pooled from all investigated participants without mood disorders or with BD. On beads digestion and capillary-liquid chromatography-nanospray tandem mass spectrometry is described in Supplementary Materials.

### Enzyme-linked immunosorbent assays (ELISA)

IL-6 serum concentrations were analyzed using Immulite 1000 IL-6 assay (Siemens Healthcare Diagnostics, Deerfield, IL) using monoclonal murine anti-IL-6 antibody (Siemens Healthcare Diagnostics, Deerfield, IL, LK6P).

Human IgG autoantibodies to oxidized low-density lipoproteins (oxLDL) in serum were measured using an Anti-Oxidized LDL (oLAB) ELISA Kit (Biomedica, Vienna, Austria).

DBP concentrations in serum were analyzed using polyclonal antibodies recognizing DBP without bound actin (Alpco, Salem, NH, USA, 30–2314) and monoclonal (DVDBP0,Quantikine ELISA, R&D Systems, Minneapolis, MN, USA) anti-DBP ELISA assays.

### Western blot

The plasma from all patients was diluted (1:60) to achieve the linear range for detection in western blot. The standard serum was prepared using a pool of equal volume (5 μL) from all tested plasma samples under reducing conditions. Serum samples were separated on 10% polyacrylamide gel under reducing conditions. Each western blot included standard plasma as well as plasma samples from two non-mood control, two MDD, and two BD patients. DBP was detected using rabbit monoclonal anti-DBP antibody (ab8130730 Abcam, Cambridge, MA, USA) and secondary infrared antibodies (LI-COR Biosciences Lincoln, NE, USA). Membranes were analyzed using an Odyssey Infrared Imaging System and ImageJ software. Data were normalized across membranes using the standard. Standard was prepared using equal aliquots from each participant from all three groups.

Additional western blots were performed using gradient polyacrylamide gel (4–20% Mini-PROTEAN #4561096, BioRad, Hercules, CA, USA) using original, deglycosylated/desialylated, plasma samples as well as plasma samples depleted of albumin and immunoglobulins. Albumin was removed from randomly selected individual serum samples using Pierce albumin/IgG removal kit (89875, Thermo Fisher Scientific, Waltham, MA, USA,) following the manufacturer’s protocol for human serum albumin and immunoglobulin removal. One-hundred and sixty microliters of PBS was added to each sample to create a final dilution of 1:32. Samples were then analyzed using western blot or were treated with protein deglycosylation mix II (P6044S, New England Biolabs, Ipswich, MA, USA) following the manufacturer’s protocol for denaturing reaction conditions. After desialylation and deglycosylation samples were analyzed by western blot as described above.

### Multiaffinity chromatography

Pooled plasma from participants with and without BD was purified on HU-6 multiaffinity removal column (4.6 × 50 mm, P.N 5185–5084, Agilent Technologies) using manufacturer’s protocol. Fractions were collected and analyzed using dot blot. DBP was detected using antibody and procedures described in western blot.

### Statistical analysis

Data was shown as mean ± standard deviation (SD). Two-tailed *t*-tests, *R* values, ANOVA one-way analysis and Tukey’s honest significance test (Tukey HSD) were used to compare differences between groups (unless otherwise stated). Normality was measured using Shapiro–Wilk Test. Correlation between normally and non-normally distributed variables was examined using Pearson’s test and Spearman Correlation, respectively. The level of significance was set at *p* < 0.05.

## Results

**Fig. 1:**
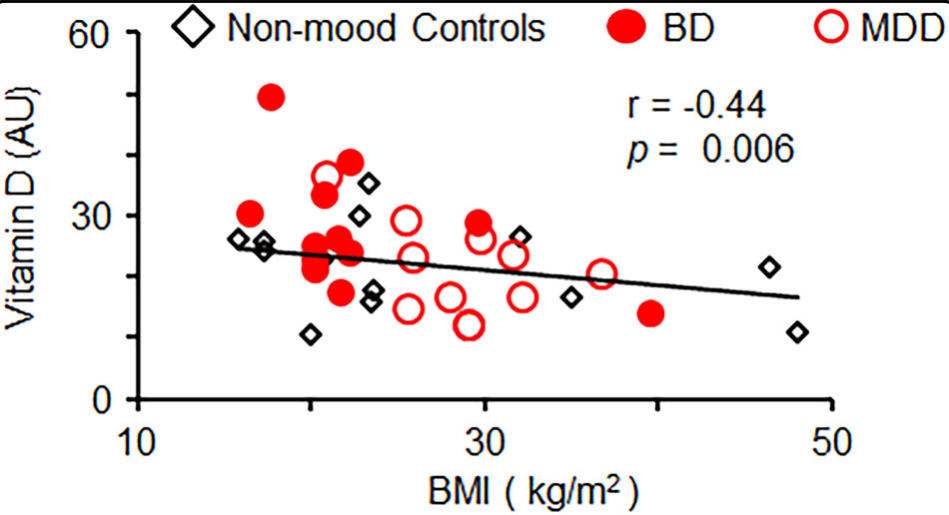
Serum 25-hydroxy vitamin D levels are inversely correlated with BMI in combined groups. Inverse linear correlation between vitamin D concentrations in plasma and BMI measured in combined groups of patients with and without MMD. Pearson correlation

BMI is described to account for contribution of adipose tissue to proinflammatory cytokine production^53^. BMI was significantly lower in BD participants (Table 1, *p* < 0.02) compared to the MDD group. BMI in non-mood controls was not significantly different from other groups. Similar changes in BMI values have been reported in other studies among adolescent MMD participants compared to adolescents without mood disorders^54,55^. As expected, BMI is positively associated with IL-6 concentration in serum from all participants (Table 2, *p* < 0.02). Also consistent with previous studies^56–58^, we found that vitamin D concentrations in serum are inversely associated with BMI in all participants (Fig. 1, *p* < 0.006). In agreement with known immune functions of vitamin D^56^, serum vitamin D concentrations showed inverse associations when compared to inflammatory cytokine IL-6 (Table 2, *p* < 0.04) and autoantibodies to oxLDL, a marker of oxidative stress (Table 2, *p* < 0.05). Thus, adolescents with and without MMD show similar associations between obesity assessed by BMI, inflammation, and vitamin D status as documented previously in other populations^13,22,53,56,57^.

**Table 1:** Comparison of different variables in non-mood control and MMD participants

Variable	Non-mood (*n* = 13)	MDD (*n* = 11)	BD (*n* = 12)
	Mean ± s.d.	Mean ± s.d.	Mean ± s.d.
Age (years)	14 ± 2.42	14.09 ± 1.22	13.9 ± 2.02
BMI (kg/m^2^)	26.59 ± 10.65	28.57 ± 4.19	22.71 ± 6.17*^,a^
Vitamin D (AU)	21.9 ± 7.26	21.01 ± 7.63	27.59 ± 9.59
IL-6 (AU)	4.71 ± 1.84	3.99 ± 0.22	3.95 ± 0.16
NFκB activation (AU)	85.64 ± 18.49	92.26 ± 37.55	135.27 ± 88.58

Data are shown as mean ± standard deviation (s.d.). One-way ANOVA set at *p* < 0.05 was used for group comparison. Asterisk indicates significant difference, the remaining differences among groups were not significant

^a^Group comparison between BD group with MMD group, *f*-ratio value is 6.28. The *p*-value is 0.022

**Table 2:** Correlation matrix of variables

Variables	Vitamin D (AU)	BMI (kg/m^2^)	oxLDL (AU)	IL-6 (AU)	NFκB (AU)
	*r* (*p*)	*r *(*p*)	*r *(*p*)	*r *(*p*)	*r *(*p*)
Vitamin D (AU)					
BMI (kg/m^2^)	−0.44 (**0.006**)				
oxLDL (AU)	−0.34 (**0.05**)	−.30 (.09)			
IL-6 (AU)	−0.35 **(0.04**)	0.29 (**0.02**)	−0.061 (0.73)		
NFκB (AU)	0.098(0.57)	−0.21(.21)	0.26 (0.12)	−0.10 (0.56)	

Correlations of variables based on two-tailed significance were measured by Pearson test in cohort of patients with and without MMD. Pearson correlation coefficient (*r*) and significance (*p*-values are shown in parenthesis, bold values indicates significant *p*-values) are shown

We also examined whether cytokines, vitamin D, and NFκB are altered in serum isolated from participants with and without MMD. We found no difference between serum vitamin D concentrations in participants from non-mood control, MMD, or BD groups (Table 1). Levels of IL-6 and autoantibodies to oxidized LDL were variable within groups and were not statistically different between participants (Table 1). Similarly, NFκB activation in reporter cells stimulated by serum from patients with and without MMD was not different among groups (Table 1). Thus, none of the major inflammatory factors or vitamin D had the power to detect differences in small groups of adolescent participants with and without MMD.

We also hypothesized that MMD pathogenesis may depend on specific inflammatory factor(s) that share properties and structural homology with inflammatory cytokines in the brain. GMFβ is an established neurotrophic and inflammatory factor implicated in activation of glia and development of the nervous system, angiogenesis, and immune function^59,60^. Using anti-GMFβ antibodies and the bait strategy^61,62^, we investigated whether serum from participants with and without MMD contains different amount of GMFβ or its protein homolog. We performed immunoprecipitation using anti-GMFβ antibodies in pooled serum from patients with and without MMD. The sequence of bound proteins to GMFβ was identified by mass spectrometry (Supplementary Table 1). Individual sample analysis by western blot showed that the weight of homologous protein was approximately 50 kD in plasma of participants with and without MMD. Next, we compared for structural homology of proteins to GMFβ. The low molecular weight vitamin D-binding protein (DBP-L, 53KD) showed 37% shared homology with GMFβ. DBP-L was present at eightfold higher levels in serum of participants with MMD compared to those without mood disorder. The structural proteins vitronectin, clusterin, and tubulins were also ~50 kD proteins homologous to GMFβ and present at higher levels in participants with MMD compared to the control group. However, in this paper we investigated DBP, given its roles in protection of nervous tissue by removing structural proteins from circulation, delivery of vitamin D, and regulation of inflammation^42^.

**Fig. 2:**
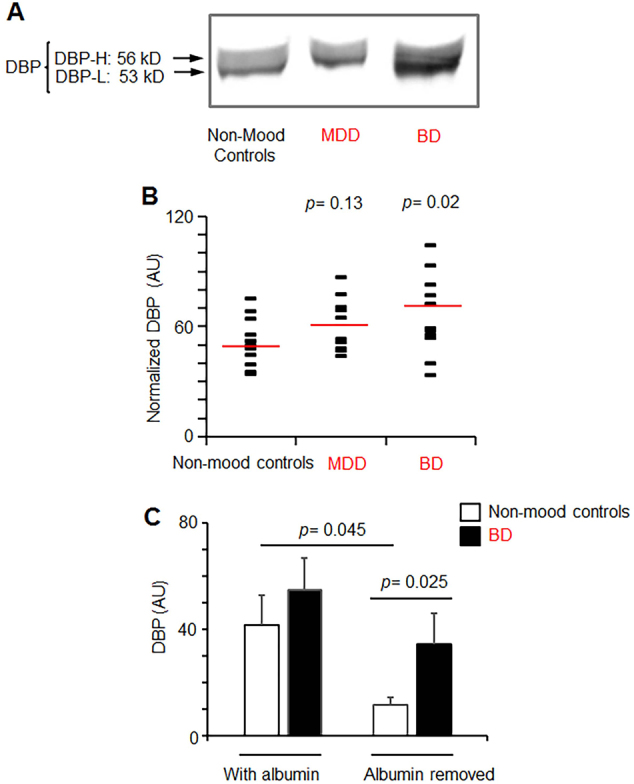
Increased DBP levels in participants with BD. **a** Representative western blot image shows total DBP in plasma from randomly selected participants without MMD (non-mood control), with MDD, and with BD. High and low molecular weight portions of heterogenous DBP are indicated by arrows (DBP-H and DBP-L, respectively). The separation was performed using 10% polyacrylamide gel. **b** Total DBP levels were quantified based on western blot analysis in serum obtained from participants in control, MMD, and BD groups. Each blot contained control, BD, MDD, and standard. Standard comprised of equal aliquots from plasma of all participants. DBP was quantified based on the density of bands and normalized across membranes using standard (arbitrary density of the standard was set as 100% in each blot). Lines represent the values obtained from individual patients after normalization by standard. Red lines show the mean value in each group. Group comparison was measured using ANOVA one-way analysis. **c** Four randomly selected plasma samples from patients with and without BD were purified from albumin and analyzed using western blot before and after albumin purification

Given the qualitative nature of immunoprecipitation/sequencing, we validated and quantified the levels of DBP in serum of participants with and without MMD using western blot, mono- and polyclonal ELISA. Western blot revealed heterogenous bands (53–56 kD) including DBP-H and DBP-L, respectively in non-mood controls, MDD, and BD (Fig. 2a). The comparison of total DBP levels in serum measured by western blot showed a moderate increase in total DBP (121%) in MDD vs. non-mood controls (Fig. 2b, *p* = 0.13, n.s.). In the serum of BD participants total DBP (137%) levels were significantly increased compared to those in non-mood control group (Fig. 2b, *p* < 0.02). The immunoprecipitation using anti-DBP antibodies revealed a similar (156%) increase in DBP serum pooled from all participants with BD compared to those without this disease (Supplementary Table 2).

The removal of albumin augmented the differences between DBP in plasma from participants with and without BD (Fig. 2c). However, this procedure impedes quantitative analysis because a substantial portion of DBP is eluted with albumin (Supplementary Fig. 1) due to the structural homology of these proteins^42^. To examine if DBP heterogeneity is a result of its modification, we performed enzymatic delycosylation and desialylation of plasma from randomly selected participants with and without BD plasma. Gradient gel electrophoresis separated DBP in plasma as a single band (Supplementary Fig. 2A). However, the treatment with deglycosylation and desialylation enzymes produced multiple modified DBP forms in plasma from both patients with and without BD (Supplementary Fig. 2B). Thus, in this pilot study, elevated total DBP levels comprising of all modifications were associated with BD.

**Fig. 3:**
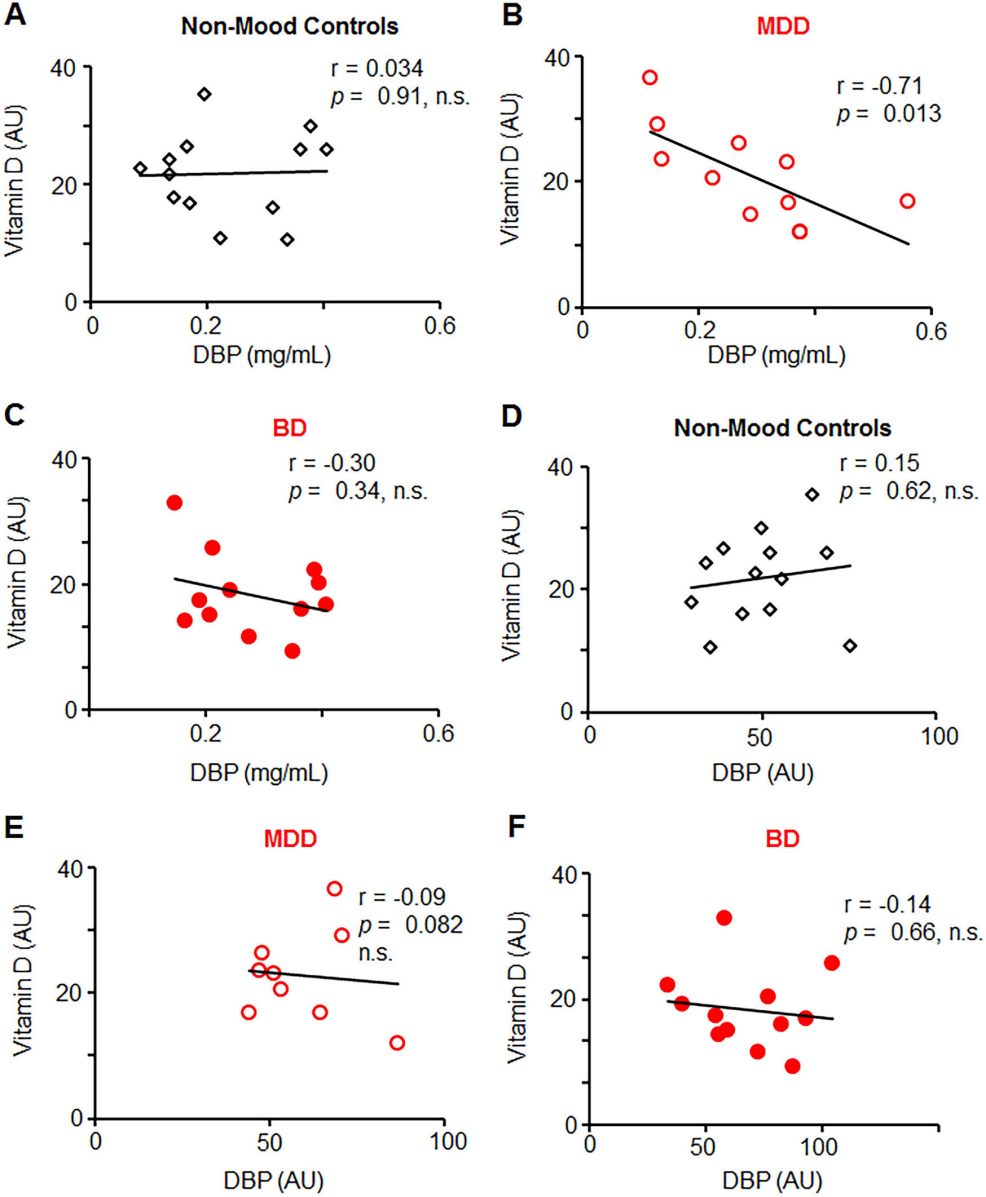
Different associations between serum vitamin D levels and DBP measured by polyclonal anti-DBP ELISA or western blot in study groups with and without MMD. Vitamin D (25-hydroxy vitamin D) levels were measured in serum by HPLC. **a**–**c** Linear correlation between vitamin D and DBP measured by polyclonal ELISA in participants without MMD (non-mood controls) (**a**) and in those with MDD (**b**), and in those with BD (**c**). **d**–**f** Linear correlation between vitamin D and DBP measured by western blot in participants without MMD (non-mood controls) (**d**) and in those with MDD (**e**), and in those with BD (**f**). Significance was examined using Pearson test, *p* > 0.05 was not significant

The heterogeneity of DBP modifications has profound impact on the outcome of DBP quantification using different methods. DBP measured by western blot was not associated with DBP measured by either monoclonal or polyclonal ELISA, suggesting that ELISA antibodies do not recognize all DBP modifications (Table 3, Supplementary Fig. 3A, B). DBP measured by monoclonal ELISA also showed no correlation with vitamin D concentration in serum in non-mood control (Fig. 3a). However, in MDD participants, but not in BD participants DBP levels recognized by polyclonal ELISA were inversely associated with vitamin D levels (Fig. 3b, *p* < 0.013 vs. Fig. 3c). Indirectly, this opposite trend suggests a change in DBP structure responsible for vitamin D transport in the MDD group compared to non-mood participants. It is expected, because DBP binds various ligands, including structural proteins, complement factors, vitamin D vitamers, and lipids^42^. DBP measured by western blot shows no significant associations with vitamin D levels in plasma (Fig. 3d–f**)** in agreement with evidence of vitamin D transport by approximately 2% of total DBP^42^. Thus, compared to western blot, ELISA appears to recognize only specific fractions of DBP that were not different among participants with and without MMD (Supplementary Figure 3C, D)

**Table 3:** Correlation matrix of variables in non-mood control, BD, and MDD groups

		Non-mood	MDD	BD
		*r *(*p*)	*r *(*p*)	*r *(*p*)
Monoclonal DBP (ng/mL)	DBP	0.36 (0.22)	−0.23 (0.55)	−0.36 (0.36)
Polyclonal DBP (ng/mL)	DBP	0.43 (0.14)	−0.40 (0.29)	−0.13 (0.70)
NFκB (AU)	DBP	0.40 (0.20)	0.15 (0.70)	−0.13 (0.70)
Vitamin D (AU)	DBP	0.17 (0.54)	−0.02 (0.97)	−0.18 (0.59)
BMI (kg/m^2^)	DBP	0.18 (0.55)	−0.27 (0.49)	0.20 (0.54)
IL-6 (AU)	DBP	0.22 (0.46)	0.43 (0.24)	−0.39 (0.21)
oxLDL (AU)	DBP	0.11 (0.75)	−0.15 (0.70)	0.01 (0.77)

Correlations of variables based on two-tailed significance are shown in a cohort of patients with and without MMD. Spearman correlation correlation coefficient (*r*) and significance (*p* < 0.05) are shown

## Discussion

The major finding in our study is the identification of increased levels of DBP in participants with BD compared to non-mood controls. The increased levels of DBP in the circulation of adolescents with BD could be interpreted in two ways: DBP plays a role in the pathogenesis of BD in adolescents or DBP is a factor associated with this disorder.

Vitamin D has been recognized in playing an important role in reducing inflammation through immunomodulatory properties^56^. However, only 2% of DBP functions in the capacity of a vitamin D transporter^42^. Moreover, vitamin D–DBP complex has a restricted passage through the blood–brain barrier^63^. Another, non-vitamin D carrier role for DBP is the binding of alternative cargo ligands in response to cellular damage and subsequent activation of inflammation^43^. Cellular and neurocellular damage leads to release of actin^43^, and possibly other structural proteins^64^. Binding of actin to GMFβ^65^ in the brain and to DBP in the cerebrospinal fluid and circulation prevents actin polymerization, aggregation, and activation of the coagulation cascade. In multiple sclerosis, DBP concentrations in the cerebrospinal fluid serve as a biomarker for this disease^32,66^. Recent investigations demonstrate that defects in actin polymerization may also be an underlying course of BD pathology^67,68^, whereas mutations in β-tubulin underlie axonal damage^69^. Moreover, stabilization of the cytoskeleton is a major basis for lithium, valproate, and paliperidone therapies^67,68^. Additionally, transcriptome analysis of post-mortem brain tissues from participants with and without schizophrenia or BD also highlights the cytoskeleton as a central deranged pathway in these disorders^70^. In our immunoprecipitation experiments, we found 35-fold increased levels of actin and other structural proteins such as tubulins, actin, actinin, or multimerin in serum from participants with BD vs. non-mood controls. However, it remains to be investigated whether DBP binds these structural proteins, or whether structural proteins were recognized by anti-DBP antibody nonspecifically in serum from participants with BD compared to non-mood controls (Supplemental Tables 1 and 2). These structural proteins required testing as candidate markers for BD in addition to DBP. In previous studies, DBP levels appear to indicate an early pathological manifestation of diseases linked to the cytoskeleton damage as a biomarker^32,66^. BD is results in derangements of the cytoskeletons^67,68^ and elevated concentrations of DBP in BD may indicate these detrimental changes.

DBP is also closely related to inflammatory processes, which were thoroughly investigated for their role in BD and MDD in the context of major inflammatory markers such as IL-6, MCP1, and TNFα^9,12,14,21^. In our study, IL-6, showed no significant changes among the different groups. Elevated IL-6 levels in circulation accompanies many proinflammatory conditions including obesity^71^. We found a significant correlation between plasma levels of IL-6 and BMI across all studied groups. These results suggest that obesity and probably other unaccounted inflammatory processes in these study participants contributed to chronic inflammation more than those activated by neurocellular damage.

DBP has a principally different mode of inflammatory action responding to early changes in damaged cellular membranes^32^ or to extracellular actin^72^. DBP binds lysophosphotidylcholine (Lyso-Pc) with a high affinity^73,74^. Lyso-Pc is a lipid mediator of acute and chronic inflammation in peripheral organs and nervous system in response to oxidative damage, inflammation, extracellular actin or changes in the intracellular actin network^72,75^. Binding of lyso-PC in the presence of T and B cells activates cleavage of DBP to DBP-L^74^, which can then activate macrophages in various diseases (DBP-MAF reviewed in ref. ^42^). In our study, we found significantly elevated DBP levels in participants with BD compared to non-mood controls, using western blot recognizing multiple forms of DBP. In contrast, ELISA using polyclonal anti-DBP antibodies, appear to recognize only DBP forms associated with decreased vitamin D transport in MDD participants. Similar prognostic changes were reported in patients with multiple sclerosis^66^. Classic conditions associated with inflammation including IL-6 levels in plasma, NFκB activation, or obesity were similar in participants with and without MMD in our study. These findings suggest DBP as a more suitable marker of BD pathology than inflammatory cytokines. More studies also need to investigate binding partners of DBP, especially lipid mediators, leading to its cleavage in BD. It additionally remains unclear whether the DBP-L molecule is a macrophage-activating factor augmenting inflammation and pathogenesis of BD. Other proteins immunoprecipitated with anti-GMFβ or anti-DBP should also be studied as potential biomarkers of MDD. Despite of these unknowns, in this pilot investigation, DBP appears to significantly change in response to BD pathogenesis and needs to be tested as a candidate marker for BD in a large cohort of adolescents and adults in the future.

The important methodological outcome of our study is the direct comparison of different tools for DBP measurement, including monoclonal and polyclonal anti-DBP based ELISA, proteomic and western blot analysis. We found that total DBP comprising of DBP-H and DBP-L forms can only be detected with western blot and proteomics. The binding of different cargo molecules to DBP^42,43^ in participants with and without MDD changes the affinity to antibodies in ELISA that obscure measurements. Reducing conditions in western blot lead to dissociation of cargo molecules from DBP and allow detection of heterogenous DBP-L and DBP-H modifications. Similar ELISA paradoxes have been reported in other studies^76^. DBP was significantly lower in African Americans vs. Caucasians when measured using a monoclonal antibody-based ELISA, but higher with a polyclonal anti-DBP ELISA^76^. In contrast, groups applying proteomic analysis to study cerebrospinal fluid from patients with multiple sclerosis found two modifications of DBP^66^ that were changed in opposite direction in multiple sclerosis patient with a different clinical course, suggesting them as new candidate prognostic biomarkers of disease aggressiveness. Our study, suggests that conclusive data on DBP function can be obtained only using proteomics and/or western blot methods.

Distinguishing BD from unipolar MDD early in the onset of illness can lead to more appropriately specific intervention, which has the potential to improve quality of life, reduce psychosocial morbidities, and recurrent mood episodes. The latter convey increased risk for suicide and for physical comorbidities, especially in adolescent patients^4,50^. Genetic, dietary, and inflammatory factors influence the pathogenesis of BD and development of the CNS^77^. DBP integrates responses to neuronal cell death, activation of inflammatory cascade, and vitamin D and lipid status^40–43,49,66,74,78,79^. Thus, DBP holds promise as a diagnostic biomarker changing in response to all major factors contributing to pathogenesis of BD, and may shed light on BD pathophysiologic mechanisms.

## Disclaimer

The content is solely the responsibility of the authors and does not necessarily represent the official views of the National Center for Research Resources or the National Institutes of Health.

## Electronic supplementary material

Supplementary materials 7 13 2017.docx

## References

[1] Lewinsohn PM, Rohde P, Seeley JR (1998). Major depressive disorder in older adolescents: prevalence, risk factors, and clinical implications. Clin. Psychol. Rev..

[2] Weissman MM (1996). Cross-national epidemiology of major depression and bipolar disorder. JAMA.

[3] Gore FM (2011). Global burden of disease in young people aged 10-24 years: a systematic analysis. Lancet.

[4] Goldstein BI (2015). Major depressive disorder and bipolar disorder predispose youth to accelerated atherosclerosis and early cardiovascular disease: A scientific statement from the American Heart Association. Circulation.

[5] Stensland MD, Schultz JF, Frytak JR (2010). Depression diagnoses following the identification of bipolar disorder: costly incongruent diagnoses. BMC Psychiatry.

[6] Zarate CA (2003). Regulation of cellular plasticity cascades in the pathophysiology and treatment of mood disorders: role of the glutamatergic system. Ann. N. Y. Acad. Sci..

[7] Watkins CC, Sawa A, Pomper MG (2014). Glia and immune cell signaling in bipolar disorder: insights from neuropharmacology and molecular imaging to clinical application. Transl. Psychiatry.

[8] Drevets WC, Price JL, Furey ML (2008). Brain structural and functional abnormalities in mood disorders: implications for neurocircuitry models of depression. Brain Struct. Funct..

[9] Munkholm K, Brauner JV, Kessing LV, Vinberg M (2013). Cytokines in bipolar disorder vs. healthy control subjects: a systematic review and meta-analysis. J. Psychiatr. Res..

[10] Lim R., Zaheer A. in *Handbook of Neurochemistry and Molecular Neurobiology: Neuroactive Proteins and Peptides* (eds Lajtha A. & Lim R) 203–222 (Springer US, 2006).

[11] Rapaport MH, Manji HK (2001). The effects of lithium on ex vivo cytokine production. Biol. Psychiatry.

[12] Brietzke E, Kapczinski F (2008). TNF-alpha as a molecular target in bipolar disorder. Prog. Neuropsychopharmacol. Biol. Psychiatry.

[13] Miller AH, Maletic V, Raison CL (2009). Inflammation and its discontents: the role of cytokines in the pathophysiology of major depression. Biol. Psychiatry.

[14] Muneer A (2016). Bipolar disorder: role of inflammation and the development of disease biomarkers. Psychiatry Investig..

[15] Tuglu C, Kara SH, Caliyurt O, Vardar E, Abay E (2003). Increased serum tumor necrosis factor-alpha levels and treatment response in major depressive disorder. Psychopharmacol. (Berl.)..

[16] Carvalho LA (2013). Lack of clinical therapeutic benefit of antidepressants is associated overall activation of the inflammatory system. J. Affect. Disord..

[17] Hiles SA, Baker AL, de Malmanche T, Attia J (2012). A meta-analysis of differences in IL-6 and IL-10 between people with and without depression: exploring the causes of heterogeneity. Brain Behav. Immun..

[18] Khairova RA, Machado-Vieira R, Du J, Manji HK (2009). A potential role for pro-inflammatory cytokines in regulating synaptic plasticity in major depressive disorder. Int. J. Neuropsychopharmacol..

[19] Tsao CW, Lin YS, Chen CC, Bai CH, Wu SR (2006). Cytokines and serotonin transporter in patients with major depression. Prog. Neuropsychopharmacol. Biol. Psychiatry.

[20] Kim YK, Jung HG, Myint AM, Kim H, Park SH (2007). Imbalance between pro-inflammatory and anti-inflammatory cytokines in bipolar disorder. J. Affect. Disord..

[21] O’Brien SM, Scully P, Scott LV, Dinan TG (2006). Cytokine profiles in bipolar affective disorder: focus on acutely ill patients. J. Affect. Disord..

[22] Miklowitz DJ (2016). Inflammatory cytokines and nuclear factor-kappa B activation in adolescents with bipolar and major depressive disorders. Psychiatry Res..

[23] Wieck A (2013). Differential neuroendocrine and immune responses to acute psychosocial stress in women with type 1 bipolar disorder. Brain. Behav. Immun..

[24] Barbosa IG (2013). Altered intracellular signaling cascades in peripheral blood mononuclear cells from BD patients. J. Psychiatr. Res..

[25] Keri S, Szabo C, Kelemen O (2014). Blood biomarkers of depression track clinical changes during cognitive-behavioral therapy. J. Affect. Disord..

[26] Pace TW (2006). Increased stress-induced inflammatory responses in male patients with major depression and increased early life stress. Am. J. Psychiatry.

[27] Jacka FN (2011). A prospective study of diet quality and mental health in adolescents. PLoS ONE.

[28] Jacka FN (2010). Associations between diet quality and depressed mood in adolescents: results from the Australian Healthy Neighbourhoods Study. Aust. N. Z. J. Psychiatry.

[29] Jacka FN, Rothon C, Taylor S, Berk M, Stansfeld SA (2013). Diet quality and mental health problems in adolescents from East London: a prospective study. Soc. Psychiatry Psychiatr. Epidemiol..

[30] Oddy WH (2009). The association between dietary patterns and mental health in early adolescence. Prev. Med..

[31] O’Neil A (2014). Relationship between diet and mental health in children and adolescents: a systematic review. Am. J. Public. Health.

[32] Yang M (2013). Vitamin D-binding protein in cerebrospinal fluid is associated with multiple sclerosis progression. Mol. Neurobiol..

[33] Adzemovic MZ, Zeitelhofer M, Hochmeister S, Gustafsson SA, Jagodic M (2013). Efficacy of vitamin D in treating multiple sclerosis-like neuroinflammation depends on developmental stage. Exp. Neurol..

[34] Gianforcaro A, Hamadeh MJ (2014). Vitamin D as a potential therapy in amyotrophic lateral sclerosis. CNS Neurosci. Ther..

[35] Sepehrmanesh Z (2016). Vitamin D supplementation affects the beck depression inventory, insulin resistance, and biomarkers of oxidative stress in patients with major depressive disorder: A randomized, controlled clinical trial. J. Nutr..

[36] Gracious BL, Finucane TL, Friedman-Campbell M, Messing S, Parkhurst MN (2012). Vitamin D deficiency and psychotic features in mentally ill adolescents: a cross-sectional study. BMC Psychiatry.

[37] Sikoglu EM (2015). Vitamin D3 supplemental treatment for mania in youth with bipolar spectrum disorders. J. Child Adolesc. Psychopharmacol..

[38] Harms LR, Eyles DW, McGrath JJ, Mackay-Sim A, Burne TH (2008). Developmental vitamin D deficiency alters adult behaviour in 129/SvJ and C57BL/6J mice. Behav. Brain Res..

[39] Eyles DW, Smith S, Kinobe R, Hewison M, McGrath JJ (2005). Distribution of the vitamin D receptor and 1 alpha-hydroxylase in human brain. J. Chem. Neuroanat..

[40] Jirikowski GF, Kaunzner UW, Dief Ael E, Caldwell JD (2009). Distribution of vitamin D binding protein expressing neurons in the rat hypothalamus. Histochem. Cell Biol..

[41] Cooke NE, McLeod JF, Wang XK, Ray K (1991). Vitamin D binding protein: genomic structure, functional domains, and mRNA expression in tissues. J. Steroid Biochem. Mol. Biol..

[42] Delanghe JR, Speeckaert R, Speeckaert MM (2015). Behind the scenes of vitamin D binding protein: more than vitamin D binding. Best Pract. Res. Clin. Endocrinol. Metab..

[43] Gomme PT, Bertolini J (2004). Therapeutic potential of vitamin D-binding protein. Trends Biotechnol..

[44] Kroksveen A. C. et al. Cerebrospinal fluid proteome comparison between multiple sclerosis patients and controls. Acta Neurol. Scand. Suppl. 2012:90–96. 10.1111/ane.12029 23278663

[45] Swamy N, Ray R (2008). Fatty acid-binding site environments of serum vitamin D-binding protein and albumin are different. Bioorg. Chem..

[46] Meier U, Gressner O, Lammert F, Gressner AM (2006). Gc-globulin: roles in response to injury. Clin. Chem..

[47] Trujillo G, Kew RR (2004). Platelet-derived thrombospondin-1 is necessary for the vitamin D-binding protein (Gc-globulin) to function as a chemotactic cofactor for C5a. J. Immunol..

[48] Kisker O (2003). Vitamin D binding protein-macrophage activating factor (DBP-maf) inhibits angiogenesis and tumor growth in mice. Neoplasia.

[49] Yamamoto N, Homma S (1991). Vitamin D3 binding protein (group-specific component) is a precursor for the macrophage-activating signal factor from lysophosphatidylcholine-treated lymphocytes. Proc. Natl Acad. Sci. USA.

[50] Goldstein BI (2015). Inflammatory markers among adolescents and young adults with bipolar spectrum disorders. J. Clin. Psychiatry.

[51] Findling RL (2010). Characteristics of children with elevated symptoms of mania: the Longitudinal Assessment of Manic Symptoms (LAMS) study. J. Clin. Psychiatry.

[52] Shen Q (2014). Adipocyte reporter assays: application for identification of anti-inflammatory and antioxidant properties of mangosteen xanthones. Mol. Nutr. Food Res..

[53] Vendrell J (2004). Resistin, adiponectin, ghrelin, leptin, and proinflammatory cytokines: relationships in obesity. Obes. Res..

[54] Correll CU (2007). Weight gain and metabolic effects of mood stabilizers and antipsychotics in pediatric bipolar disorder: a systematic review and pooled analysis of short-term trials. J. Am. Acad. Child Adolesc. Psychiatry.

[55] Krishnan KR (2005). Psychiatric and medical comorbidities of bipolar disorder. Psychosom. Med..

[56] Mutt SJ, Hypponen E, Saarnio J, Jarvelin MR, Herzig KH (2014). Vitamin D and adipose tissue-more than storage. Front. Physiol..

[57] Parikh SJ (2004). The relationship between obesity and serum 1,25-dihydroxy vitamin D concentrations in healthy adults. J. Clin. Endocrinol. Metab..

[58] Wortsman J, Matsuoka LY, Chen TC, Lu Z, Holick MF (2000). Decreased bioavailability of vitamin D in obesity. Am. J. Clin. Nutr..

[59] Aldoori A. D. *Elucidation of Signaling Mediators between Adipose and Neural Tissue*. The Ohio State University (2014).

[60] Khawaja X, Xu J, Liang JJ, Barrett JE (2004). Proteomic analysis of protein changes developing in rat hippocampus after chronic antidepressant treatment: Implications for depressive disorders and future therapies. J. Neurosci. Res..

[61] Mann M, Hendrickson RC, Pandey A (2001). Analysis of proteins and proteomes by mass spectrometry. Annu. Rev. Biochem..

[62] Tezel G (2013). A proteomics view of the molecular mechanisms and biomarkers of glaucomatous neurodegeneration. Prog. Retin. Eye Res..

[63] Pardridge WM, Sakiyama R, Coty WA (1985). Restricted transport of vitamin D and A derivatives through the rat blood-brain barrier. J. Neurochem..

[64] Lee SA (2011). Construction and analysis of the protein-protein interaction networks for schizophrenia, bipolar disorder, and major depression. BMC Bioinforma..

[65] Goroncy AK (2009). NMR solution structures of actin depolymerizing factor homology domains. Protein Sci.: a Publ. Protein..

[66] Perga S (2015). Vitamin D binding protein isoforms and apolipoprotein E in cerebrospinal fluid as prognostic biomarkers of multiple sclerosis. PLoS. One..

[67] Calabrese B, Halpain S (2014). Lithium prevents aberrant NMDA-induced F-actin reorganization in neurons. Neuroreport.

[68] Corena-McLeod M (2013). New model of action for mood stabilizers: phosphoproteome from rat pre-frontal cortex synaptoneurosomal preparations. PLoS ONE.

[69] Niwa S, Takahashi H, Hirokawa N (2013). beta-Tubulin mutations that cause severe neuropathies disrupt axonal transport. Embo. J..

[70] Zhao Z (2015). Transcriptome sequencing and genome-wide association analyses reveal lysosomal function and actin cytoskeleton remodeling in schizophrenia and bipolar disorder. Mol. Psychiatry.

[71] Yudkin JS, Kumari M, Humphries SE, Mohamed-Ali V (2000). Inflammation, obesity, stress and coronary heart disease: is interleukin-6 the link?. Atherosclerosis.

[72] Dubose DA, Shepro D, Hechtman HB (1989). Modulation of phospholipase A2 lytic activity by actin and myosin. Inflammation.

[73] White P, Cooke N (2000). The multifunctional properties and characteristics of vitamin D-binding protein. Trends Endocrinol. Metab..

[74] Yamamoto N, Kumashiro R, Yamamoto M, Willett NP, Lindsay DD (1993). Regulation of inflammation-primed activation of macrophages by two serum factors, vitamin D3-binding protein and albumin. Infect. Immun..

[75] Guillemot-Legris O (2016). High-fat diet feeding differentially affects the development of inflammation in the central nervous system. J. Neuroinflamm..

[76] Aloia J (2015). Free 25(OH)D and the vitamin D paradox in African Americans. J. Clin. Endocrinol. Metab..

[77] Paus T, Keshavan M, Giedd JN (2008). Why do many psychiatric disorders emerge during adolescence?. Nat. Rev. Neurosci..

[78] Chun RF (2012). New perspectives on the vitamin D binding protein. Cell Biochem. Funct..

[79] Garcion E, Wion-Barbot N, Montero-Menei CN, Berger F, Wion D (2002). New clues about vitamin D functions in the nervous system. Trends Endocrinol. Metab..

