# Supplementary material for: Bipolar disorder in youth is associated with increased levels of vitamin D-binding protein
Source: Transl Psychiatry. 2018 Mar 13;8:61. doi: 10.1038/s41398-018-0109-7 (PMC5847532; doi:10.1038/s41398-018-0109-7)
Supplement: Supplementary file 1 — Supplementary materials 7 13 2017.docx [file 41398_2018_109_MOESM1_ESM.docx]

Supplementary Information

Supplemental Experimental Procedures, Supplementary Tables 1, 2, Supplementary Figures 1-3 with Legends, Disclosures.

Supplementary Experimental Procedures

**Methods**

***Vitamin D detection***

Vitamin D analysis on serum was performed at the Esoterix Laboratory Services, Inc. (Austin, Texas). Serum proteins were precipitated and 25-hydroxycalciferol and 25-hydroxyergocalciferol were extracted from serum samples with methanol. These metabolites were measured in these extracts by high-pressure liquid chromatography (HPLC) coupled with mass spectrometry detection. Calibration curves were generated by spiking known amounts of purified 25-hydroxycalciferol and 25-hydroxyergocalciferol into charcoal stripped human serum. The amount of each metabolite in every sample was calculated using duplicate calibration curves.

***NFκB assay***

The cumulative NFκB-activation potential of plasma was measured using NF-κB/Green fluorescence protein (GFP) biosensor assay. This assay was developed in a mouse 3T3-L1 preadipocyte cell line that was stably transfected with a NF-κB-RE/GFP lentiviral reporter vector and an mCherry lentiviral control vector as described previously^50^. Reporter cells were stimulated with 20 ng/mL lipopolysaccharide (LPS) serving as a positive control (100%). 1% patient serum was used to stimulate NFκB-reporter cells for 48 hours. Then cells were lysed with a RIPA buffer. GFP fluorescence was measured in 80 μL of cell lysates at wavelengths Ex/Em 485/528 and mCherry at Ex/Em 587/640 using a Synergy H1 Hybrid Multi-Mode Microplate Reader (BioTek, Winooski, VT, USA). NFκB-activation was measured as a ratio of GFP to mCherry fluorescence. Experiments performed on different days were normalized to the standard plasma, which was prepared using equal volume (5μL) from all tested plasma samples. Fluorescence values were normalized to values of the standard plasma. Identification of coded participant disorder status was released after the experiments and data analyses were completed.

***Immunoprecipitation***

A non-denaturing buffered lysate (20mM Tris pH 8.0, 50mM NaCl, 0.5% IGEPAL) was pre-cleared to reduce non-specific binding and background. A 100 μL aliquot of 50% slurry Dynabead (Invitrogen) was added to 1 mL of the lysate. The lysate solution was incubated for 30 min at 4°C with gentle agitation. Afterward, the beads were magnetically captured and the solution removed. The captured beads were washed and conjugated with a 50 μL of off-target bovine control IgG for 30 minutes at 4°C. Beads were magnetically captured, washed, and added to the cleared lysate to incubate for 30 minutes at 4°C with gentle agitation. The bead IgG control conjugate was removed and the beads discarded.

A 1μg aliquot of mouse anti-glia maturation factor beta (GMFβ)-antibody (Proteintech, Rosemont, IL, USA) was mixed per 50μg of pre-cleared lysate and allowed to bind overnight in a cold room. Fresh beads were washed and applied to the antibody-lysate mixture, and allowed to bind for 30 minutes at 4°C. The beads were magnetically captured and washed 5x with a gentle wash buffer (20mM Tris pH 8.0, 50mM NaCl, 0.05% IGEPAL) and 5x with buffer sans detergent (20mM Tris pH 8.0, 50mM NaCl). The final wash was not removed awaiting protein digestions. Cell lysates were incubated with GMFβ-antibodies-conjugated beads and according to manufacturers’ protocol (Millipore, Darmstadt, Germany). Proteins bound to GMFβ-antibodies-conjugated beads were eluted, digested and identified by LC/MSMS on a Thermo Fisher Fusion mass spectrometer coupled with a Dionex U3000 RSLC system. ***LTQ Orbitrap***

Capillary-liquid chromatography-nanospray tandem mass spectrometry (Capillary-LC/MS/MS) of global protein identification was performed on a Thermo Fisher LTQ orbitrap mass spectrometer equipped with a microspray source (Michrom Bioresources Inc, Auburn, CA) operated in positive ion mode. Samples were separated on a capillary column (0.2X150mm Magic C18AQ 3µ 200A, Bruker Daltonics) using an UltiMate™ 3000 HPLC system from Thermo Scientific. Each sample was injected into the µ-Precolumn Cartridge (Thermo Scientific) and desalted with 50 mM acetic acid for 5 minutes. The injector port was then switched to inject and the peptides were eluted off of the trap onto the column. Mobile phase A was 50mM acetic acid in water and acetonitrile was used as mobile phase B. Flow rate was set at 2 µl/min. Mobile phase B was increased from 2% to 35% in 35 min, then increased from 35%-50% in 5 min and then increase again from 55% to 85% in 2 min. Mobile phase B was kept at 85% for another 2 min before being brought back quickly to 2% in 0.1 min. The column was equilibrated at 2% of mobile phase B (or 98% A) for 15 min before the next sample injection. MS/MS data was acquired with a spray voltage of 2.2 KV and a capillary temperature of 175 °C is used. The scan sequence of the mass spectrometer was based on the preview mode data dependent TopTen™ method: the analysis was programmed for a full scan recorded between m/z 350 – 2000 and a MS/MS scan to generate product ion spectra to determine amino acid sequence in consecutive scans of the five most abundant peaks in the spectrum. To achieve high mass accuracy MS determination, the full scan was performed at FT mode and the resolution was set at 60,000. The AGC Target ion number for FT full scan was set at 1 x 106 ions, maximum ion injection time was set at 1000 ms and micro scan number was set at 1. MSn was performed using ion trap mode to ensure the highest signal intensity of MSn spectra. The AGC Target ion number for ion trap MSn scan was set at 10000 ions, maximum ion injection time was set at 50 ms and micro scan number was set at 1. The CID fragmentation energy was set to 35%. Dynamic exclusion is enabled with a repeat count of 1 within 12 s, a mass list size limit of 500, exclusion duration of 8 s and a low mass width and high mass width of 30 ppm. When the sample is digested with trypsin, an exclusion list containing major trypsin autolysis peptides is applied so these peaks will not be selected for fragmentation. The reject mass width window is set at 30 ppm.

Sequence information from the MS/MS data was processed by converting the raw files into a merged file (.mgf) using an in-house program, RAW2MZXML_n_MGF_batch (merge.pl, a Perl script). Isotope distributions for the precursor ions of the MS/MS spectra were deconvoluted to obtain the charge states and monoisotopic m/z values of the precursor ions during the data conversion. The resulting mgf files were searched using Mascot Daemon by Matrix Science version 2.3.2 (Boston, MA) and the database searched against the most recent SwissProt database version 2012_06 (536,489 sequences; 190,389,898 residues) or NCBI databases version 20120515 (18,099,548 sequences; 6,208,559,787 residues. The mass accuracy of the precursor ions was set to 20 ppm, accidental pick of 1 13C peaks was also included into the search. The fragment mass tolerance was set to 0.5 Da. Considered variable modifications were oxidation (Met), deamidation (N and Q) and carbamidomethylation (Cys). Four missed cleavages for the enzyme were permitted. A decoy database was also searched to determine the false discovery rate (FDR) and peptides were filtered according to the FDR. The significance threshold was set at p<0.05 and bold red peptides is required for valid peptide identification. Proteins with a Mascot score of 50 or higher with a minimum of two unique peptides from one protein having a -b or -y ion sequence tag of five residues or better were accepted. Any modifications or low score peptide/protein identifications were manually checked for validation. 126 proteins were identified by immunoprecipitation using anti-GMFβ monoclonal antibody that were present at higher levels in serum from MMD than non-mood control group (**Supplementary Table 1**). We excluded proteins that were not present in plasma in one group of patients (Ratio 0 or n/a). Abundant plasma proteins such as proteins, complement factors, and immunoglobulins as well as structural intracellular were excluded from analysis of homology. The remaining protein structures were analyzed for the homology to GMFβ (Accession number NP_004115.1) using NCBI BLAST program. VDBP accession number used for this program was AAA61704.1.

**Digestion of proteins bound anti DBP antibodies conjugated on beads.**

Beads were washed with 50 mM ammonium bicarbonate (30-50uL each time) three times. Each time, the supernatant were kept and pooled. After the third wash, 5uL of DTT (5ug/uL in50 mM ammonium bicarbonate) is added and the sample is incubated at 56C for 15 min. After the incubation, 5uL of iodoacetamide (15 mg/ml in 50mM ammonium bicarbonate) is added and the sample is kept in dark at room temperature for 30 min. Sequencing grade-modified trypsin (Promega, Madison WI) prepared in 50 mM ammonium bicarbonate was added to the sample with an estimation of 1:20 /1:100 enzyme-substrate ratio and the reaction was carried on at 37C for overnight. The reaction is quenched the next morning by adding acetic acid for acidification. Supernatant was concentrated for LC/MSMS analysis.

**Capillary-liquid chromatography-nanospray tandem mass spectrometry (Capillary-LC/MS/MS)**

Global protein identification was performed on a Thermo Fisher Fusion mass spectrometer equipped with a Thermo Easy-Spary operated in positive ion mode. Samples were separated on a Thermo Nano C18 column using an UltiMate™ 3000 HPLC system from Thermo Scientific. Each sample was injected into the µ-Precolumn Cartridge (Thermo Scientific,) and desalted with 50 mM acetic acid for 5 minutes. The injector port was then switched to inject and the peptides were eluted off of the trap onto the column. Mobile phase A was 0.1% formic acid in water and acetonitrile was used as mobile phase B. Flow rate was set at 300 nl/min. Typically, mobile phase B was increased from 2% to 10% in 15 min and then increased from 10%-30% in 105 min and then increased again from 30%-55% in another 30 min. Mobile phase B was again increased from 55%-95% in 3 min and kept at 95% for another 2 min before being brought back quickly to 2% in 1 min. The column was equilibrated at 2% of mobile phase B (or 98% A) for 10 min before the next sample injection. MS/MS data was acquired with a spray voltage of 1.7 KV and a capillary temperature of 275 °C is used; S-Lens RF level was set at 60%. The scan sequence of the mass spectrometer was based on the preview mode data dependent TopSpeed™ method with CID and ETD as fragmentation methods: the analysis was programmed for a full scan recorded between m/z 400 – 1600 and a MS/MS scan to generate product ion spectra to determine amino acid sequence in consecutive scans of the five most abundant peaks in the spectrum. To achieve high mass accuracy MS determination, the full scan was performed at FT mode and the resolution was set at 120,000. The AGC Target ion number for FT full scan was set at 4 x 105 ions, maximum ion injection time was set at 50 ms and micro scan number was set at 1. MSn was performed using ion trap mode to ensure the highest signal intensity of MSn spectra. The AGC Target ion number for ion trap MSn scan was set at 100 ions, maximum ion injection time was set at 250 ms and micro scan number was set at 1. The CID fragmentation energy was set to 35%. Dynamic exclusion is enabled with a repeat count of 1, exclusion duration of 60s and a low mass width and high mass width of 10ppm.

Sequence information from the MS/MS data was processed by converting the .raw files into a merged file (.mgf) using MSConvert (proteoWizard). The resulting mgf files were searched using Mascot Daemon by Matrix Science version 2.5.1 (Boston, MA) and the database searched against the most recent SwissProt or NCBI databases. The mass accuracy of the precursor ions were set to 10ppm, accidental pick of 1 13C peaks was also included into the search. The fragment mass tolerance was set to 0.5 Da. Considered variable modifications were oxidation (Met), deamidation (N and Q) and carbamidomethylation (Cys). Four missed cleavages for the enzyme were permitted. A decoy database was also searched to determine the false discovery rate (FDR) and peptides were filtered according to the FDR. The significance threshold was set at p<0.05 and bold red peptides is required for valid peptide identification.

### *Western blot*

The plasma from all patients was diluted (1:60) to achieve the linear range for detection in western blot. The standard serum was prepared using a pool of equal volume (5μL) from all tested plasma samples under reducing conditions. Serum samples were separated on 10% polyacrylamide gel under reducing conditions using Mini-PROTEAN Tetra cell (Bio-Rad, Hercules, CA, USA) at 4°C and then transferred to a polyvinylidene fluoride membrane (Immobilon-P; Millipore, Darmstadt, Germany). Each western blot included standard plasma as well as plasma samples from 2 non-mood control, 2 MDD, and 2 BD patients. All samples were measured using 8 western blots. DBP protein expression was detected using a primary rabbit monoclonal to DBP antibody (Abcam, Cambridge, MA, USA, ab81307) at a 1:1000 dilution in an Odyssey blocking buffer (LI-COR Biosciences Lincoln, NE, USA) and secondary infrared antibodies (LI-COR Biosciences Lincoln, NE, USA) at 1:50,000 dilution in an Odyssey blocking buffer. Membranes were scanned and analyzed using an Odyssey Infrared Imaging System. Band densities of the high and low molecular weight DBP were quantified using ImageJ software and normalized across membranes using the standard.

***Statistical Analysis***

Data was shown as mean + standard deviation of the mean. Two-tailed t-tests, R^2^ values, ANOVA one-way analysis and Tukey’s honest significance test (Tukey HSD) were used to compare differences between groups (unless otherwise stated). Correlation between normally and non-normally distributed variables was examined using Pearson’s test and Spearman Correlation, respectively. Normality was measured using Shapiro-Wilk Test. The level of significance was set at *p* < 0.05 for these exploratory analyses.

**Supplementary Table 1: Proteins identified using immunoprecipitation with anti GMFβ antibodies of pooled plasma from participants with and without MMD.** Proteins present at higher than 130% ratio in plasma from BD vs. non-mood (NM) control participants are shown. Bold font indicates proteins with a molecular weight of approximately 50kD. This molecular weight was detected in plasma from individual participants with and without MMD using western blot and the same antibodies (data are not shown).

| # | | Identified Proteins (126) | Accession N | MW, kDa | MMD | NM  control | Ratio | Homology |
| --- | --- | --- | --- | --- | --- | --- | --- | --- |
| 1 | Vitamin D-binding protein | | AAA61704.1 | **53** | 57 | 7 | 8.1 | 37% |
| 2 | Tubulin alpha-4A chain | | P68366.1 | **50** | 103 | 8 | 12.9 | 29% |
| 3 | Vitronectin | | EAW51082.1 | **54** | 18 | 3 | 6.0 | 83% |
| 4 | Clusterin | | P10909.1 | **52** | 13 | 4 | 3.3 | 56% |
| 5 | Tubulin beta-1 | | NP_110400.1 | **50** | 106 | 1 | 106.0 | 21% |
| 6 | Tubulin beta chain | | P07437.2 | **50** | 83 | 1 | 83.0 | 33% |
| 7 | Talin-1 | | NP_006280.3 | 270 | 344 | 4 | 86.0 | 43% |
| 8 | Filamin-A | | BAJ83965.1 | 281 | 540 | 5 | 108.0 | 38% |
| 9 | Multimerin-1 | | AAH63848.1 | 138 | 79 | 2 | 39.5 | 41% |
| 10 | Myosin-9 | | NP_002464.1 | 227 | 1242 | 32 | 38.8 | 38% |
| 11 | Actin, cytoplasmic 1 | | NP_001092.1 | 42 | 207 | 6 | 34.5 | N/A |
| 12 | Coagulation factor V | | AAB59401.1 | 252 | 40 | 3 | 13.3 | 45% |
| 13 | Latent-transforming growth  factor beta-binding protein 1 | | Q14766.4 | 187 | 24 | 3 | 8.0 | 63% |
| 14 | Myosin light polypeptide 6 | | P60660.2 | 17 | 69 | 6 | 11.5 | 25% |
| 15 | Thrombospondin-1 | | AAI36470.1 | 129 | 290 | 34 | 8.5 | 52% |
| 16 | Apolipoprotein B-100 | | P04114.2 | 516 | 71 | 23 | 3.1 | 32% |
| 17 | Complement C1q subcomponent subunit A | | C1QA | 26 | 687 | 264 | 2.6 |  |
| 18 | Ig heavy chain V-III region GAL | | HV320 | 13 | 4 | 2 | 2.0 |  |
| 19 | Serum albumin | | ALBU | 69 | 103 | 48 | 2.1 |  |
| 20 | Complement C1q subcomponent subunit B | | C1QB | 27 | 136 | 65 | 2.1 |  |
| 21 | Keratin, type II cytoskeletal 1 | | K2C1 | 66 | 71 | 38 | 1.9 |  |
| 22 | CD5 antigen-like | | CD5L | 38 | 11 | 6 | 1.8 |  |
| 23 | Keratin, type I cytoskeletal 10 | | K1C10 | 59 | 45 | 27 | 1.7 |  |
| 24 | Complement C3 | | CO3 | 187 | 29 | 18 | 1.6 |  |
| 25 | Ig mu chain C region | | IGHM | 49 | 103 | 68 | 1.5 |  |
| 26 | Immunoglobulin J chain | | IGJ | 18 | 3 | 2 | 1.5 |  |
| 27 | Ig gamma-3 chain C region | | IGHG3 | 41 | 420 | 309 | 1.4 |  |

**Supplementary Table 2: Proteins identified using immunoprecipitation with anti DBP antibodies of pooled plasma from participants with BD and non MMD controls (NM-Control).** Proteins present at higher than 130% ratio in plasma from BD vs. non-mood (NM) control participants are shown. PMID is provided for references describing binding of thrombospondin 1 to DBP, cytoskeleton proteins altered in response to vitamin D, or proteins associated with DBP polymorphism.

| **Identified Proteins (166)** | **Accession Number** | **IP_NM**  **Control** | **IP_BD** | **Ratio, BD/NM**  **Contr** | **PMID** |
| --- | --- | --- | --- | --- | --- |
| Vitamin D-binding protein | VTDB | 23 | 37 | 1.56 |  |
| ***COAGULATION CASCADE PROTEINS*** | |  |  |  |  |
| Complement component C8 alpha chain | CO8A | 3 | 7 | 2.72 |  |
| Complement C1q subcomponent subunit A | C1QA | 6 | 15 | 2.53 |  |
| Complement C1q subcomponent subunit C | C1QC | 3 | 11 | 3.06 |  |
| Complement C1q subcomponent subunit B | C1QB | 11 | 15 | 1.36 |  |
| Complement C1s subcomponent | C1S | 4 | 7 | 1.63 |  |
| Complement component C6 | CO6 | 3 | 4 | 1.36 |  |
| Platelet factor 4 | PLF4 | 1 | 5 | 5.45 |  |
| **Thrombospondin-1** | TSP1 | 13 | 25 | 1.91 | 15356163 |
| von Willebrand factor | VWF | 2 | 4 | 2.04 |  |
| Coagulation factor V | FA5 | 1 | 2 | 2.72 |  |
| Plasma kallikrein | KLKB1 | 3 | 5 | 1.36 |  |
| Antithrombin-III | ANT3 | 7 | 11 | 1.53 |  |
| Plasminogen | PLMN | 11 | 17 | 1.47 |  |
| Vitronectin | VTNC | 8 | 11 | 1.36 |  |
| ***STRUCTURAL PROTEINS*** | |  |  |  |  |
| Tubulin alpha-4A | TBA4A | 6 | 8 | 1.36 | 2168775 |
| Tubulin alpha-1C chain | TBA1C | 4 | 7 | 1.63 | 2168775 |
| Tubulin beta-1 chain | TBB1 | 7 | 9 | 1.36 | 2168775 |
| Gelsolin | GELS | 7 | 13 | 1.87 |  |
| Alpha-actinin-1 | ACTN1 | 3 | 5 | 1.36 | 2168775 |
| Multimerin-1 | MMRN1 | 5 | 7 | 1.36 |  |
| Integrin alpha-IIb | ITA2B | 7 | 13 | 1.87 |  |
| Integrin beta-3 | ITB3 | 5 | 11 | 2.04 |  |
| Transgelin-2 | TAGL2 | 3 | 7 | 2.04 |  |
| Fermitin family homolog 3 | URP2 | 3 | 5 | 1.36 |  |
| ***BINDING PROTEINS*** |  |  |  |  |  |
| Corticosteroid-binding globulin | CBG | 2 | 2 | 1.36 |  |
| Protein | AMBP | 7 | 9 | 1.36 |  |
| Retinol-binding protein 4 | RET4 | 3 | 4 | 1.36 | 26962819 |
| Zinc-alpha-2-glycoprotein | ZA2G | 3 | 5 | 1.82 |  |
| Fetuin-B | FETUB | 2 | 4 | 2.04 |  |
| Kininogen-1 | KNG1 | 9 | 14 | 1.63 |  |
| ***PROTEINS REGULATING OXIDATIVE STRESS & OTHER STRESS RESPONSES*** | | | | |  |
| Carboxypeptidase B2 | CBPB2 | 2 | 2 | 1.36 |  |
| Glutathione peroxidase 3 | GPX3 | 3 | 4 | 1.36 |  |
| Peroxiredoxin-6 | PRDX6 | 1 | 2 | 2.72 |  |
| Adenylyl cyclase-associated protein 1 | CAP1 | 1 | 2 | 2.72 |  |
| Phosphatidylinositol-glycan-specific phospholipase D | PHLD | 1 | 2 | 2.72 |  |
| ***APOPROTEINS*** |  |  |  |  |  |
| Beta-2-glycoprotein 1 | APOH | 3 | 9 | 3.63 |  |
| Apolipoprotein C-II | APOC2 | 2 | 5 | 2.72 |  |
| Apolipoprotein(a) | APOA | 15 | 53 | 3.6 |  |
| Apolipoprotein C-III | APOC3 | 5 | 11 | 2.04 |  |
| Apolipoprotein A-IV | APOA4 | 10 | 18 | 1.7 |  |
| Apolipoprotein E | APOE | 25 | 34 | 1.36 |  |
|  |  |  |  |  |  |

**
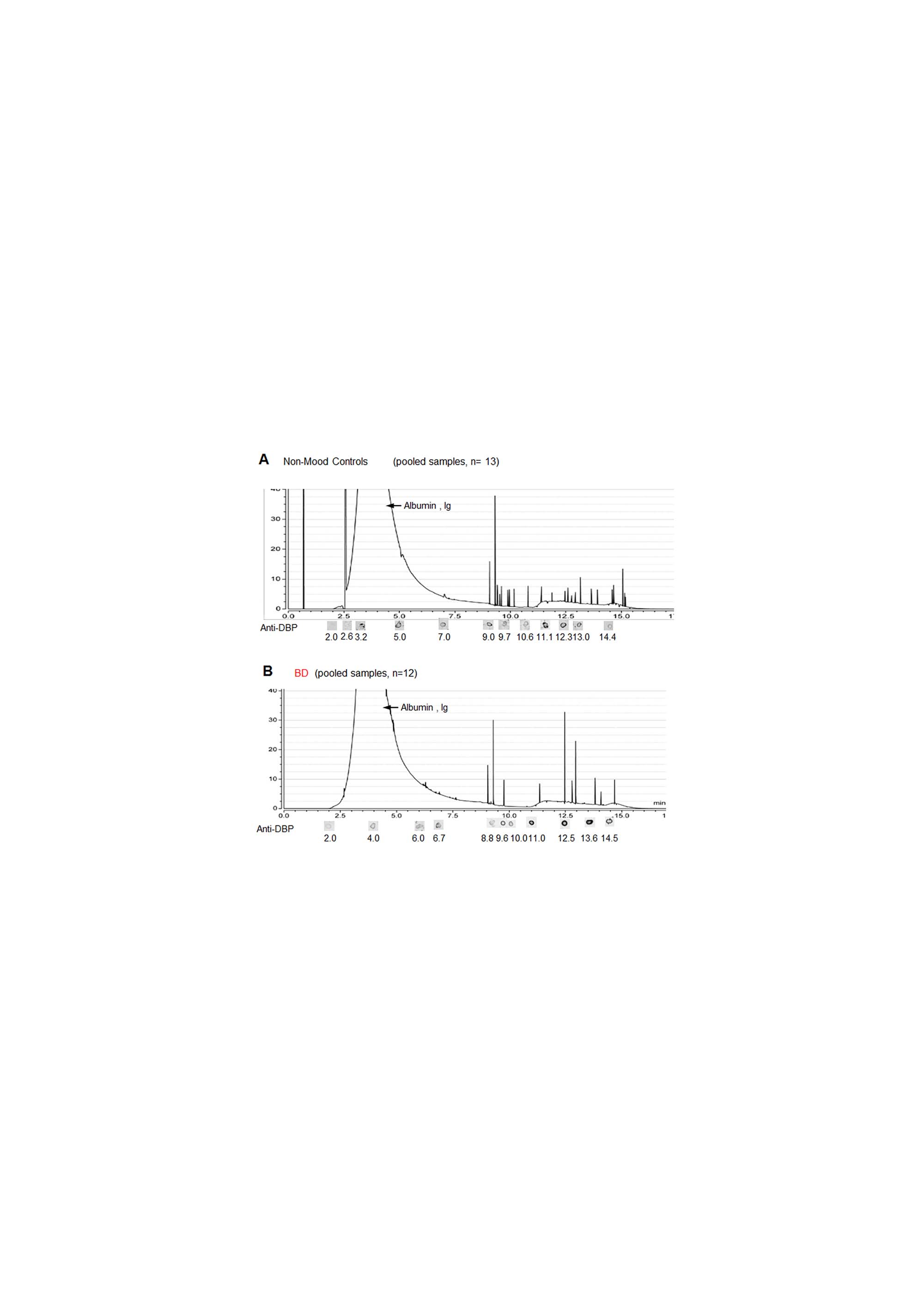
**

**Supplementary Figure 1. Representative chromatograms and DBP levels in fraction separated from plasma pooled from participants with and without BD.**

Equal aliquots (100μL) were pooled from plasma isolated from participants without BD (Non-mood controls) and participants with BD. Pooled plasma samples were separated using Multiple Affinity Removal System fractionating proteins from human plasma. Fractions were collected and DBP levels were analyzed using dot blot. Each spot contains same fraction volumes (10 μL). Fractions were collected at time intervals indicated under each dot blot.

**
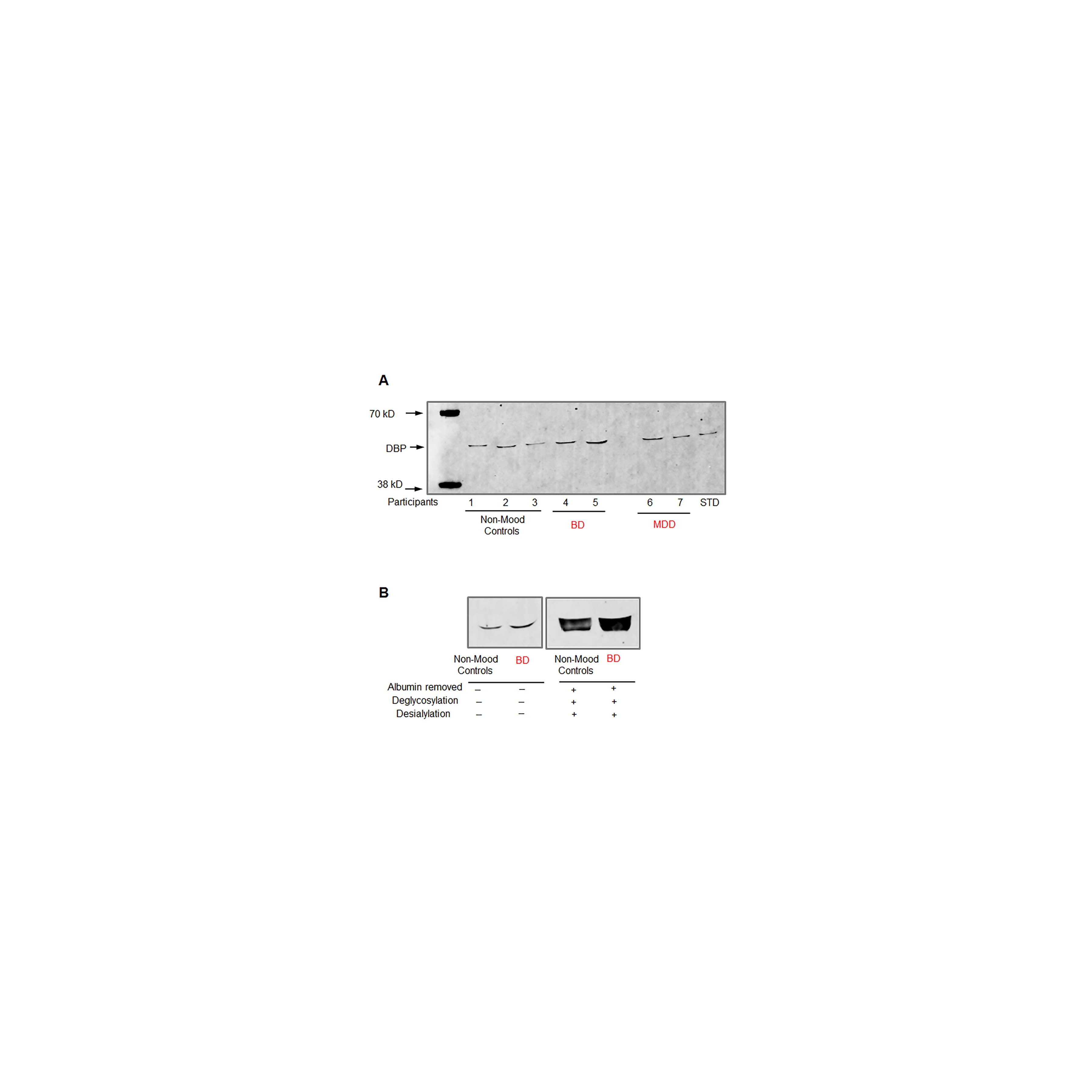
**

**Supplementary Figure 2. DBP levels in plasma from participants with and without BD using gradient electrophoresis with and without purification of albumin, desialylation and deglycosylation.**

(A) Representative western blot image shows total DBP in plasma from randomly selected participants without MMD (non-mood control), with MDD, and with BD. The separation was performed using 4-20% polyacrylamide gel. (B) Representative western blot of randomly selected plasma samples from patients with and without BD before and after purification from albumin, and enzymatic desialylation and deglycosylation. This treatment increase heterogeneity of DBP. The separation was performed using 4-20% polyacrylamide gel.

**
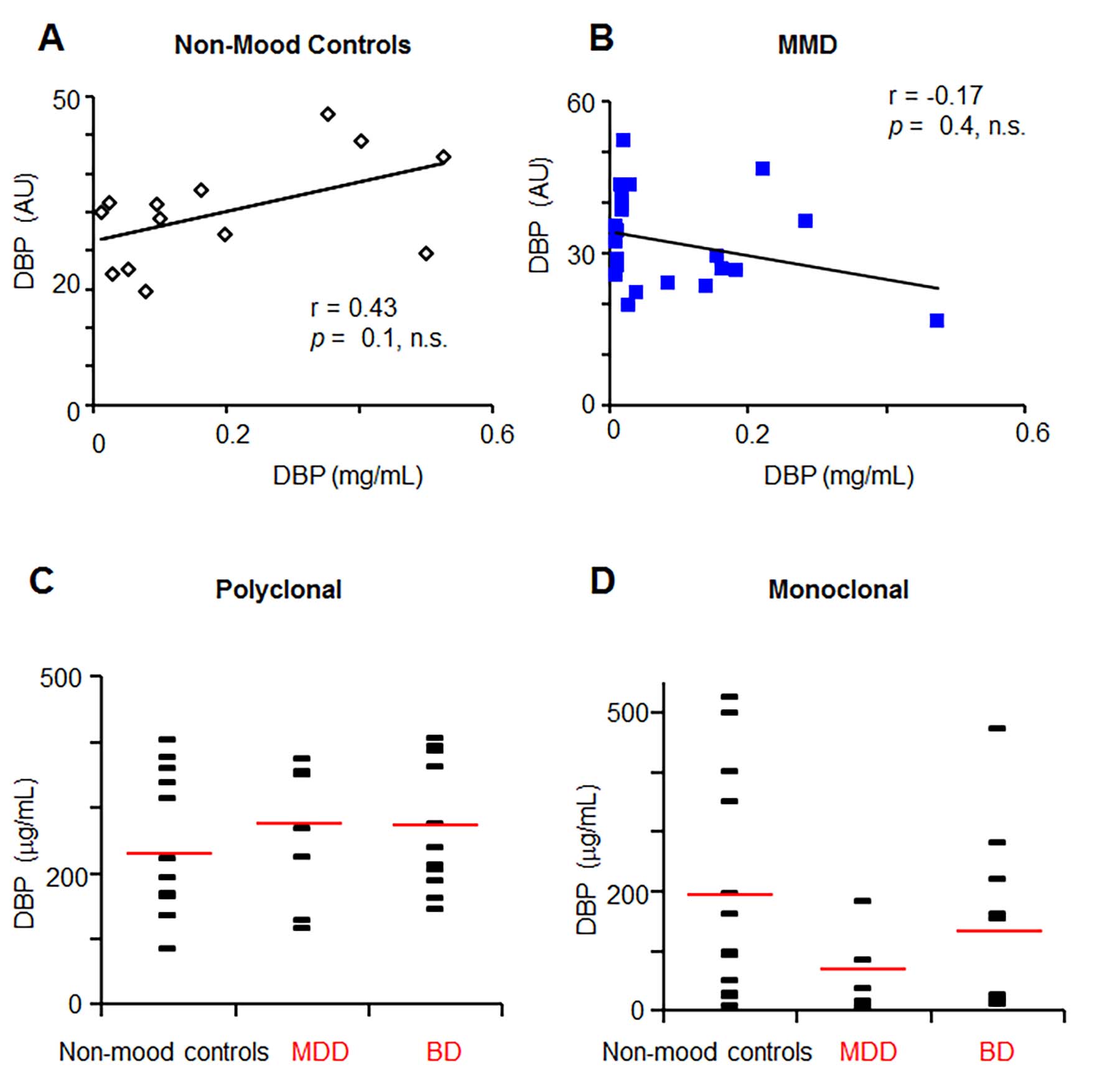
**

**Supplementary Figure 3. Different association between DBP measured by western blot (DBP, AU) and DBP measured by monoclonal ELISA (DBP mg/ml) in study groups with and without MMD**

(A,B) Linear correlations between DBP measured by western blot and DBP measured by ELISA in same patients from a control (A) and MMD (B) groups. AU, arbitrary units. Significance was examined using Pearson test, *p*>0.05 were not significant (n.s.). (C,D) Total DBP levels were quantified in serum obtained from participants in control, MMD, and BD groups using polyclonal (C) and monoclonal (D) anti-DBP ELISA respectively. Lines represent the values obtained from individual patients. Red lines show the mean value in each group. Group comparison measured using ANOVA one-way analysis showed *p*>0.05, n.s.

**DISCLOSURES:**

Dr. Arnold has received research funding from Curemark, Forest, Lilly, Neuropharm, Novartis, Noven, Shire, Supernus, and Young Living (as well as NIH and Autism Speaks) and has consulted with or been on advisory boards for Arbor, Gowlings, Ironshore, Neuropharm, Novartis, Noven, Organon, Otsuka, Pfizer, Roche, Seaside Therapeutics, Sigma Tau, Shire, Tris Pharma, and Waypoint and received travel support from Noven. Dr. Fristad receives: royalties from Guilford Press, American Psychiatric Press and Child & Family Psychological Services; research funding from Janssen and NIMH; and honoraria from Physicians Post-Graduate Press and the American Occupational Therapy Association. Dr. Gracious has consulted with AstraZeneca and Novo Nordisk.
